# 2,2′-Imino­diethanaminium 2,2′-(disulfanyldi­yl)dibenzoate dihydrate

**DOI:** 10.1107/S1600536810007117

**Published:** 2010-02-27

**Authors:** Grant A. Broker, Edward R. T. Tiekink

**Affiliations:** a 5959 FM 1960 Road West, Houston, Texas 77069, USA; b Department of Chemistry, University of Malaya, 50603 Kuala Lumpur, Malaysia

## Abstract

In the title hydrated salt, C_4_H_15_N_3_^2+^·C_14_H_8_O_4_S_2_^−^·2H_2_O, the dication (with both terminal –NH_2_ groups protonated) adopts a U-shaped conformation, the N_amine_—C—C—N_aza­nium_ torsion angles being 57.9 (6) and 60.3 (6)°. The dianion is twisted: the central C—S—S—C torsion angle is 81.3 (2)° and the dihedral angle between the benzene rings is 85.4 (3)°. In the crystal, a chain in the *a*-axis direction mediated by water–carboxyl­ate O—H⋯O hydrogen bonds through a sequence of alternating 12-membered {⋯OCO⋯HOH}_2_ and eight-membered {⋯O⋯HOH}_2_ synthons occurs, which involves only one of the carboxyl­ate residues. The second carboxyl­ate residue participates in N—H⋯O hydrogen bonding, generating a three-dimensional network, along with aza­nium–water N—H⋯O hydrogen bonds.

## Related literature

For related studies on co-crystal/salt formation involving 2-[(2-carboxy­phen­yl)disulfan­yl]benzoic acid, see: Broker & Tiekink (2007 ▶); Broker *et al.* (2008 ▶). For software for searching the Cambridge Structural Database, see: Bruno *et al.* (2002 ▶).
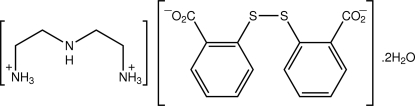


## Experimental

### 

#### Crystal data

C_4_H_15_N_3_^+^·C_14_H_8_O_4_S_2_^−^·2H_2_O
*M* *_r_* = 445.55
Triclinic, 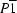

*a* = 7.804 (3) Å
*b* = 11.472 (5) Å
*c* = 12.701 (4) Å
α = 102.162 (9)°
β = 104.806 (4)°
γ = 102.776 (7)°
*V* = 1028.1 (6) Å^3^
*Z* = 2
Mo *K*α radiation
μ = 0.30 mm^−1^
*T* = 173 K
0.40 × 0.20 × 0.03 mm

#### Data collection

Rigaku AFC12/SATURN724 diffractometer
Absorption correction: multi-scan (*ABSCOR*; Higashi, 1995 ▶) *T*_min_ = 0.832, *T*_max_ = 1
9871 measured reflections
3529 independent reflections
3380 reflections with *I* > 2σ(*I*)
*R*_int_ = 0.023

#### Refinement

*R*[*F* ^2^ > 2σ(*F* ^2^)] = 0.067
*wR*(*F* ^2^) = 0.175
*S* = 1.28
3529 reflections
277 parameters
7 restraints
H-atom parameters constrained
Δρ_max_ = 0.74 e Å^−3^
Δρ_min_ = −0.39 e Å^−3^

### 

Data collection: *CrystalClear* (Rigaku/MSC, 2005 ▶); cell refinement: *CrystalClear*; data reduction: *CrystalClear*; program(s) used to solve structure: *SHELXS97* (Sheldrick, 2008 ▶); program(s) used to refine structure: *SHELXL97* (Sheldrick, 2008 ▶); molecular graphics: *ORTEPII* (Johnson, 1976 ▶) and *DIAMOND* (Brandenburg, 2006 ▶); software used to prepare material for publication: *SHELXL97*.

## Supplementary Material

Crystal structure: contains datablocks global, I. DOI: 10.1107/S1600536810007117/hb5342sup1.cif

Structure factors: contains datablocks I. DOI: 10.1107/S1600536810007117/hb5342Isup2.hkl

Additional supplementary materials:  crystallographic information; 3D view; checkCIF report

## Figures and Tables

**Table 1:** Hydrogen-bond geometry (Å, °)

*D*—H⋯*A*	*D*—H	H⋯*A*	*D*⋯*A*	*D*—H⋯*A*
N1—H1*N*⋯O1*W*^i^	0.91	2.27	2.939 (6)	130
N1—H1*N*⋯O2*W*^ii^	0.91	2.33	3.038 (6)	135
N1—H2*N*⋯O4^iii^	0.91	1.85	2.731 (7)	164
N1—H3*N*⋯O3^iv^	0.91	2.24	2.981 (6)	138
N2—H4*N*⋯O3^v^	0.88	2.21	3.069 (6)	166
N3—H5*N*⋯O1*W*^vi^	0.91	2.06	2.961 (6)	172
N3—H6*N*⋯O2*W*^vi^	0.91	1.94	2.844 (6)	170
N3—H7*N*⋯O3^iv^	0.91	1.98	2.835 (6)	156
O1w—H1*W*⋯O2	0.84	1.90	2.720 (5)	167
O1w—H2*W*⋯O2^vii^	0.84	1.99	2.803 (5)	162
O2w—H3*W*⋯O1	0.84	1.89	2.732 (6)	176
O2w—H4*W*⋯O2^vi^	0.84	1.92	2.751 (5)	171

Symmetry codes: (i) 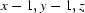; (ii) 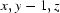; (iii) 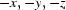; (iv) 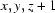; (v) 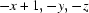; (vi) 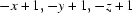; (vii) 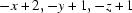.

**Fig. 1.**
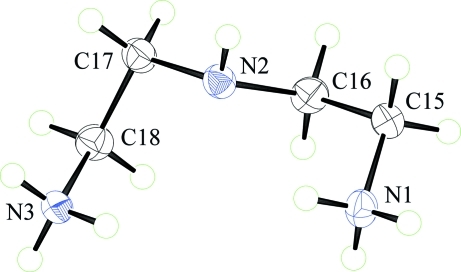
Molecular structure of the cation in (I) showing displacement ellipsoids at the 70% probability level.

**Fig. 2.**
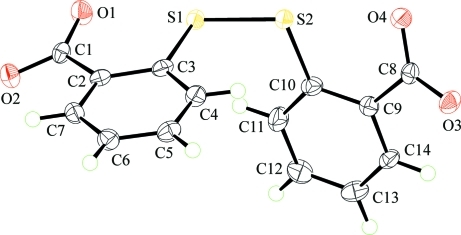
Molecular structure of the anion in (I) showing displacement ellipsoids at the 70% probability level.

**Fig. 3.**
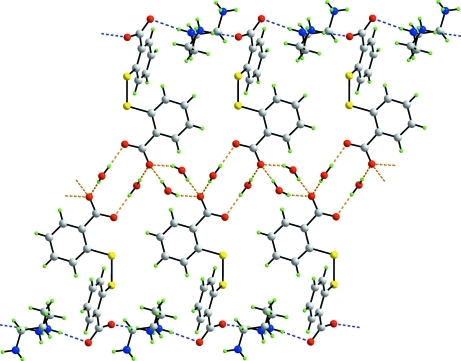
A view of the supramolcular chain in (I) mediated by O–H···O hydrogen bonding (orange dashed lines) via alternating 12-membered {···OCO···HOH}2 and eight-membered {···O···HOH}2 synthons; the second carboxylate ligand forms N–H···O (blue dashed lines) hydrogen bonds exclusively with two of these running parallel to the chain illustrated. Colour code: S, yellow; O, red; N, blue; C, grey; H, green.

**Fig. 4.**
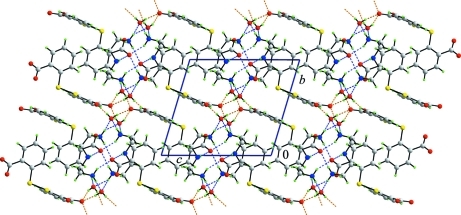
The unit cell contents of (I) viewed in projection down the a axis. The O–H···O and N–H···O hydrogen bonds are shown as orange and blue dashed lines, respectively. Colour code: S, yellow; O, red; N, blue; C, grey; H, green.

## supplementary crystallographic information

### Comment

The title salt dihydrate, (I), was obtained during crystallisation experiments involving various N-containing species with the dicarboxylic acid, 2-[(2-carboxyphenyl)disulfanyl]benzoic acid (Broker & Tiekink, 2007; Broker *et al.*, 2008). The asymmetric unit of (I) comprises an di-aminium cation, Fig. 1, a dinegative 2-[(2-carboxylatophenyl)disulfanyl]benzoate anion, Fig. 2, and two solvent water molecules of crystallisation.

Confirmation that protonation has occurred at each of the terminal primary amines is found in terms of the pattern of intermolecular interactions and consistent with this conclusion is the observation that the central-N2 amine participates in both donor and acceptor hydrogen bonding interactions (see below). The dication adopts a U-shaped conformation: the N1—C15—C16—N2 and N2—C17—C18—N3 torsion angles are 57.9 (6) and 60.3 (6) °, respectively. In accord with expectation (Broker & Tiekink, 2007), the anion is twisted, the C3–S1–S2–C10 torsion angle = 81.3 (2) °, a conformation stabilised by intramolecular S···O interactions of 2.643 (4) Å for S1···O1 and 2.724 (4) Å for S2···O4.

The water molecules in (I) play a pivotal role in the crystal packing, Table 1. As shown in Fig. 3, pairs of water molecules bridge two carboxylate residues leading to a 12-membered {···OCO···HOH}_2_ synthon. Translationally related synthons are bridged by a pair of water molecules forming eight-membered {···O···HOH}_2_ synthons and leading to a chain aligned along the *a* axis, Fig. 3. By contrast, the O3, O4-carboxylate residue does not participate in O–H···O hydrogen bonding but forms N–H···O interactions instead, involving both the aminium-N1 and amine-N2 groups. In the global crystal packing, the chains are sandwiched so that immediately above and below each row of hydrogen bonded carboxylate residues/water molecules are located aminium groups, with each of the nitrogen-bound acidic-hydrogen atoms forming a significant hydrogen bond, Table 1, as emphasized in the view of Fig. 4.

### Experimental

The title salt was obtained by dissolving 2-[(2-carboxyphenyl)disulfanyl]benzoic acid (0.100 g, Fluka) in ethanol (20 ml) to which was added the amine in 1:1, 1:2 and 1:3 stoichiometric ratios in three separate experiments. Regardless of the stoichiometry, only colurless plates of (I) were harvested as proved by multiple unit cell determinations (m.pt. 381–382 K).

### Refinement

The H-atoms located from difference maps but placed in their idealised positions (O–H = 0.84 Å, N–H = 0.88–0.91 Å, and C–H 0.95–0.99 Å) and were included in the refinement in the riding model approximation with *U*_iso_(H) set to 1.2-1.5*U*_eq_(carrier atom).

### Crystal data

**Table:**

C_4_H_15_N_3_^+^·C_14_H_8_O_4_S_2_^−^·2H_2_O	*Z* = 2
*M**_r_* = 445.55	*F*(000) = 472
Triclinic, *P*1	*D*_x_ = 1.439 Mg m^−^^3^
Hall symbol: -P 1	Mo *K*α radiation, λ = 0.71070 Å
*a* = 7.804 (3) Å	Cell parameters from 1579 reflections
*b* = 11.472 (5) Å	θ = 2.8–30.4°
*c* = 12.701 (4) Å	µ = 0.30 mm^−^^1^
α = 102.162 (9)°	*T* = 173 K
β = 104.806 (4)°	Plate, colourless
γ = 102.776 (7)°	0.40 × 0.20 × 0.03 mm
*V* = 1028.1 (6) Å^3^	

### Data collection

**Table:**

Rigaku AFC12K/SATURN724 diffractometer	3529 independent reflections
Radiation source: fine-focus sealed tube	3380 reflections with *I* > 2σ(*I*)
graphite	*R*_int_ = 0.023
ω scans	θ_max_ = 25.0°, θ_min_ = 2.8°
Absorption correction: multi-scan (*ABSCOR*; Higashi, 1995)	*h* = −9→9
*T*_min_ = 0.832, *T*_max_ = 1	*k* = −13→13
9871 measured reflections	*l* = −14→15

### Refinement

**Table:**

Refinement on *F*^2^	Primary atom site location: structure-invariant direct methods
Least-squares matrix: full	Secondary atom site location: difference Fourier map
*R*[*F*^2^ > 2σ(*F*^2^)] = 0.067	Hydrogen site location: inferred from neighbouring sites
*wR*(*F*^2^) = 0.175	H-atom parameters constrained
*S* = 1.28	*w* = 1/[σ^2^(*F*_o_^2^) + (0.0108*P*)^2^ + 6.0488*P*] where *P* = (*F*_o_^2^ + 2*F*_c_^2^)/3
3529 reflections	(Δ/σ)_max_ < 0.001
277 parameters	Δρ_max_ = 0.74 e Å^−^^3^
7 restraints	Δρ_min_ = −0.39 e Å^−^^3^

### Special details

**Table:**

Geometry. All esds (except the esd in the dihedral angle between two l.s. planes) are estimated using the full covariance matrix. The cell esds are taken into account individually in the estimation of esds in distances, angles and torsion angles; correlations between esds in cell parameters are only used when they are defined by crystal symmetry. An approximate (isotropic) treatment of cell esds is used for estimating esds involving l.s. planes.
Refinement. Refinement of *F*^2^ against ALL reflections. The weighted *R*-factor wR and goodness of fit *S* are based on *F*^2^, conventional *R*-factors *R* are based on *F*, with *F* set to zero for negative *F*^2^. The threshold expression of *F*^2^ > σ(*F*^2^) is used only for calculating *R*-factors(gt) etc. and is not relevant to the choice of reflections for refinement. *R*-factors based on *F*^2^ are statistically about twice as large as those based on *F*, and *R*- factors based on ALL data will be even larger.

### Fractional atomic coordinates and isotropic or equivalent isotropic displacement parameters (Å^2^)

**Table:**

	*x*	*y*	*z*	*U*_iso_*/*U*_eq_	
S1	0.36783 (17)	0.36668 (12)	0.05188 (10)	0.0220 (3)	
S2	0.27851 (18)	0.29462 (11)	−0.12164 (10)	0.0227 (3)	
O1	0.4707 (5)	0.4706 (4)	0.2733 (3)	0.0289 (8)	
O2	0.7336 (5)	0.4825 (3)	0.4019 (3)	0.0259 (8)	
O3	0.2557 (5)	0.0464 (3)	−0.4359 (3)	0.0269 (8)	
O4	0.1636 (5)	0.2013 (3)	−0.3519 (3)	0.0261 (8)	
O1W	1.0465 (5)	0.6687 (3)	0.5318 (3)	0.0261 (8)	
H1W	0.9510	0.6167	0.4832	0.039*	
H2W	1.1163	0.6333	0.5664	0.039*	
O2W	0.3984 (5)	0.6179 (3)	0.4457 (3)	0.0276 (8)	
H3W	0.4214	0.5702	0.3947	0.041*	
H4W	0.3596	0.5795	0.4885	0.041*	
N1	0.2010 (6)	−0.1911 (4)	0.3919 (4)	0.0261 (10)	
H1N	0.1997	−0.2594	0.4178	0.039*	
H2N	0.0846	−0.1829	0.3721	0.039*	
H3N	0.2786	−0.1219	0.4476	0.039*	
N2	0.3889 (6)	0.0195 (4)	0.3355 (4)	0.0237 (9)	
H4N	0.4998	0.0098	0.3591	0.036*	
N3	0.2433 (6)	0.2148 (4)	0.4298 (4)	0.0247 (9)	
H5N	0.1469	0.2457	0.4346	0.037*	
H6N	0.3517	0.2726	0.4752	0.037*	
H7N	0.2308	0.1443	0.4528	0.037*	
C1	0.6340 (7)	0.4671 (4)	0.3007 (4)	0.0196 (10)	
C2	0.7199 (7)	0.4399 (4)	0.2082 (4)	0.0200 (10)	
C3	0.6121 (7)	0.3884 (4)	0.0920 (4)	0.0210 (10)	
C4	0.7005 (7)	0.3573 (5)	0.0129 (4)	0.0247 (11)	
H4	0.6293	0.3201	−0.0648	0.030*	
C5	0.8917 (7)	0.3796 (5)	0.0455 (5)	0.0265 (11)	
H5	0.9492	0.3573	−0.0101	0.032*	
C6	0.9987 (7)	0.4334 (5)	0.1573 (4)	0.0249 (11)	
H6	1.1296	0.4508	0.1789	0.030*	
C7	0.9117 (7)	0.4620 (5)	0.2382 (4)	0.0224 (10)	
H7	0.9846	0.4974	0.3158	0.027*	
C8	0.2166 (6)	0.1048 (4)	−0.3544 (4)	0.0210 (10)	
C9	0.2344 (6)	0.0572 (4)	−0.2513 (4)	0.0200 (10)	
C10	0.2594 (7)	0.1319 (4)	−0.1426 (4)	0.0226 (11)	
C11	0.2712 (8)	0.0787 (5)	−0.0541 (5)	0.0320 (13)	
H11	0.2877	0.1289	0.0195	0.038*	
C12	0.2593 (9)	−0.0472 (5)	−0.0706 (5)	0.0358 (14)	
H12	0.2676	−0.0819	−0.0085	0.043*	
C13	0.2359 (7)	−0.1201 (5)	−0.1749 (5)	0.0292 (12)	
H13	0.2278	−0.2059	−0.1863	0.035*	
C14	0.2238 (6)	−0.0689 (4)	−0.2650 (4)	0.0222 (10)	
H14	0.2079	−0.1205	−0.3380	0.027*	
C15	0.2665 (7)	−0.2058 (5)	0.2913 (4)	0.0271 (11)	
H15A	0.1831	−0.2816	0.2311	0.033*	
H15B	0.3924	−0.2159	0.3125	0.033*	
C16	0.2700 (8)	−0.0929 (5)	0.2468 (4)	0.0272 (11)	
H16A	0.3167	−0.1029	0.1810	0.033*	
H16B	0.1428	−0.0858	0.2212	0.033*	
C17	0.4030 (7)	0.1345 (5)	0.3004 (5)	0.0261 (11)	
H17A	0.4037	0.1170	0.2208	0.031*	
H17B	0.5212	0.1977	0.3487	0.031*	
C18	0.2445 (7)	0.1852 (5)	0.3100 (4)	0.0275 (11)	
H18A	0.2561	0.2615	0.2845	0.033*	
H18B	0.1263	0.1230	0.2603	0.033*	

### Atomic displacement parameters (Å^2^)

**Table:**

	*U*^11^	*U*^22^	*U*^33^	*U*^12^	*U*^13^	*U*^23^
S1	0.0203 (6)	0.0241 (6)	0.0190 (6)	0.0080 (5)	0.0041 (5)	0.0012 (5)
S2	0.0293 (7)	0.0196 (6)	0.0175 (6)	0.0089 (5)	0.0039 (5)	0.0039 (5)
O1	0.0195 (18)	0.040 (2)	0.0248 (19)	0.0098 (16)	0.0064 (15)	0.0045 (16)
O2	0.0237 (18)	0.032 (2)	0.0198 (18)	0.0067 (15)	0.0056 (15)	0.0074 (15)
O3	0.036 (2)	0.0271 (19)	0.0220 (19)	0.0119 (16)	0.0149 (16)	0.0057 (15)
O4	0.032 (2)	0.0248 (19)	0.0225 (18)	0.0112 (16)	0.0082 (15)	0.0074 (15)
O1W	0.029 (2)	0.0189 (18)	0.024 (2)	0.0069 (15)	0.0024 (15)	0.0014 (15)
O2W	0.035 (2)	0.0265 (19)	0.026 (2)	0.0112 (16)	0.0148 (17)	0.0072 (16)
N1	0.032 (2)	0.021 (2)	0.025 (2)	0.0068 (19)	0.0093 (19)	0.0074 (18)
N2	0.027 (2)	0.023 (2)	0.024 (2)	0.0098 (19)	0.0102 (19)	0.0084 (18)
N3	0.026 (2)	0.021 (2)	0.031 (2)	0.0095 (18)	0.0132 (19)	0.0077 (18)
C1	0.021 (2)	0.016 (2)	0.019 (2)	0.0021 (19)	0.007 (2)	0.0035 (19)
C2	0.023 (2)	0.014 (2)	0.024 (3)	0.0073 (19)	0.008 (2)	0.0059 (19)
C3	0.029 (3)	0.017 (2)	0.023 (3)	0.011 (2)	0.012 (2)	0.008 (2)
C4	0.027 (3)	0.024 (3)	0.023 (3)	0.008 (2)	0.008 (2)	0.006 (2)
C5	0.030 (3)	0.026 (3)	0.029 (3)	0.011 (2)	0.015 (2)	0.007 (2)
C6	0.021 (3)	0.026 (3)	0.027 (3)	0.007 (2)	0.007 (2)	0.007 (2)
C7	0.022 (2)	0.022 (2)	0.021 (3)	0.004 (2)	0.004 (2)	0.007 (2)
C8	0.019 (2)	0.020 (2)	0.019 (2)	0.002 (2)	0.005 (2)	0.002 (2)
C9	0.017 (2)	0.020 (2)	0.022 (2)	0.0034 (19)	0.0053 (19)	0.006 (2)
C10	0.021 (2)	0.017 (2)	0.028 (3)	0.005 (2)	0.006 (2)	0.006 (2)
C11	0.047 (3)	0.030 (3)	0.020 (3)	0.014 (3)	0.011 (2)	0.005 (2)
C12	0.053 (4)	0.029 (3)	0.030 (3)	0.014 (3)	0.010 (3)	0.019 (3)
C13	0.031 (3)	0.020 (3)	0.042 (3)	0.008 (2)	0.016 (3)	0.012 (2)
C14	0.019 (2)	0.016 (2)	0.027 (3)	0.0043 (19)	0.006 (2)	0.000 (2)
C15	0.029 (3)	0.024 (3)	0.027 (3)	0.007 (2)	0.011 (2)	0.003 (2)
C16	0.031 (3)	0.030 (3)	0.019 (3)	0.005 (2)	0.008 (2)	0.006 (2)
C17	0.030 (3)	0.025 (3)	0.026 (3)	0.010 (2)	0.012 (2)	0.008 (2)
C18	0.030 (3)	0.030 (3)	0.027 (3)	0.010 (2)	0.011 (2)	0.011 (2)

### Geometric parameters (Å, °)

**Table:**

S1—C3	1.787 (5)	C4—H4	0.9500
S1—S2	2.0540 (18)	C5—C6	1.375 (7)
S2—C10	1.796 (5)	C5—H5	0.9500
O1—C1	1.244 (6)	C6—C7	1.393 (7)
O2—C1	1.273 (6)	C6—H6	0.9500
O3—C8	1.253 (6)	C7—H7	0.9500
O4—C8	1.262 (6)	C8—C9	1.509 (7)
O1W—H1W	0.8400	C9—C14	1.401 (7)
O1W—H2W	0.8400	C9—C10	1.403 (7)
O2W—H3W	0.8400	C10—C11	1.380 (7)
O2W—H4W	0.8400	C11—C12	1.393 (8)
N1—C15	1.484 (6)	C11—H11	0.9500
N1—H1N	0.9100	C12—C13	1.353 (8)
N1—H2N	0.9100	C12—H12	0.9500
N1—H3N	0.9100	C13—C14	1.384 (8)
N2—C16	1.455 (7)	C13—H13	0.9500
N2—C17	1.470 (7)	C14—H14	0.9500
N2—H4N	0.8800	C15—C16	1.515 (7)
N3—C18	1.491 (7)	C15—H15A	0.9900
N3—H5N	0.9100	C15—H15B	0.9900
N3—H6N	0.9100	C16—H16A	0.9900
N3—H7N	0.9100	C16—H16B	0.9900
C1—C2	1.506 (7)	C17—C18	1.500 (7)
C2—C7	1.396 (7)	C17—H17A	0.9900
C2—C3	1.419 (7)	C17—H17B	0.9900
C3—C4	1.388 (7)	C18—H18A	0.9900
C4—C5	1.391 (7)	C18—H18B	0.9900
			
C3—S1—S2	104.23 (17)	C14—C9—C10	118.2 (5)
C10—S2—S1	103.71 (18)	C14—C9—C8	118.1 (4)
H1W—O1W—H2W	111.4	C10—C9—C8	123.7 (4)
H3W—O2W—H4W	111.4	C11—C10—C9	119.1 (5)
C15—N1—H1N	109.5	C11—C10—S2	121.7 (4)
C15—N1—H2N	109.5	C9—C10—S2	119.3 (4)
H1N—N1—H2N	109.5	C10—C11—C12	121.4 (5)
C15—N1—H3N	109.5	C10—C11—H11	119.3
H1N—N1—H3N	109.5	C12—C11—H11	119.3
H2N—N1—H3N	109.5	C13—C12—C11	120.1 (5)
C16—N2—C17	114.3 (4)	C13—C12—H12	119.9
C16—N2—H4N	108.1	C11—C12—H12	119.9
C17—N2—H4N	109.8	C12—C13—C14	119.6 (5)
C18—N3—H5N	109.5	C12—C13—H13	120.2
C18—N3—H6N	109.5	C14—C13—H13	120.2
H5N—N3—H6N	109.5	C13—C14—C9	121.6 (5)
C18—N3—H7N	109.5	C13—C14—H14	119.2
H5N—N3—H7N	109.5	C9—C14—H14	119.2
H6N—N3—H7N	109.5	N1—C15—C16	110.4 (4)
O1—C1—O2	124.6 (4)	N1—C15—H15A	109.6
O1—C1—C2	118.2 (4)	C16—C15—H15A	109.6
O2—C1—C2	117.2 (4)	N1—C15—H15B	109.6
C7—C2—C3	118.9 (4)	C16—C15—H15B	109.6
C7—C2—C1	118.8 (4)	H15A—C15—H15B	108.1
C3—C2—C1	122.3 (4)	N2—C16—C15	110.1 (4)
C4—C3—C2	118.7 (5)	N2—C16—H16A	109.6
C4—C3—S1	122.1 (4)	C15—C16—H16A	109.6
C2—C3—S1	119.3 (4)	N2—C16—H16B	109.6
C3—C4—C5	121.1 (5)	C15—C16—H16B	109.6
C3—C4—H4	119.5	H16A—C16—H16B	108.2
C5—C4—H4	119.5	N2—C17—C18	111.2 (4)
C6—C5—C4	120.9 (5)	N2—C17—H17A	109.4
C6—C5—H5	119.6	C18—C17—H17A	109.4
C4—C5—H5	119.6	N2—C17—H17B	109.4
C5—C6—C7	118.8 (5)	C18—C17—H17B	109.4
C5—C6—H6	120.6	H17A—C17—H17B	108.0
C7—C6—H6	120.6	N3—C18—C17	110.3 (4)
C6—C7—C2	121.6 (5)	N3—C18—H18A	109.6
C6—C7—H7	119.2	C17—C18—H18A	109.6
C2—C7—H7	119.2	N3—C18—H18B	109.6
O3—C8—O4	124.8 (5)	C17—C18—H18B	109.6
O3—C8—C9	118.1 (4)	H18A—C18—H18B	108.1
O4—C8—C9	117.1 (4)		
			
C3—S1—S2—C10	81.3 (2)	O3—C8—C9—C10	156.8 (5)
O1—C1—C2—C7	166.2 (4)	O4—C8—C9—C10	−22.9 (7)
O2—C1—C2—C7	−15.1 (7)	C14—C9—C10—C11	−0.5 (7)
O1—C1—C2—C3	−16.1 (7)	C8—C9—C10—C11	179.0 (5)
O2—C1—C2—C3	162.6 (4)	C14—C9—C10—S2	178.7 (4)
C7—C2—C3—C4	2.6 (7)	C8—C9—C10—S2	−1.9 (7)
C1—C2—C3—C4	−175.1 (4)	S1—S2—C10—C11	10.9 (5)
C7—C2—C3—S1	−177.0 (4)	S1—S2—C10—C9	−168.2 (4)
C1—C2—C3—S1	5.3 (6)	C9—C10—C11—C12	0.2 (8)
S2—S1—C3—C4	−0.7 (4)	S2—C10—C11—C12	−178.9 (5)
S2—S1—C3—C2	178.9 (3)	C10—C11—C12—C13	0.1 (9)
C2—C3—C4—C5	−2.1 (7)	C11—C12—C13—C14	0.0 (9)
S1—C3—C4—C5	177.5 (4)	C12—C13—C14—C9	−0.3 (8)
C3—C4—C5—C6	−0.1 (8)	C10—C9—C14—C13	0.5 (7)
C4—C5—C6—C7	1.9 (8)	C8—C9—C14—C13	−178.9 (5)
C5—C6—C7—C2	−1.5 (8)	C17—N2—C16—C15	179.5 (4)
C3—C2—C7—C6	−0.8 (7)	N1—C15—C16—N2	57.9 (6)
C1—C2—C7—C6	177.0 (4)	C16—N2—C17—C18	83.9 (5)
O3—C8—C9—C14	−23.8 (7)	N2—C17—C18—N3	60.3 (6)
O4—C8—C9—C14	156.5 (4)		

### Hydrogen-bond geometry (Å, °)

**Table:**

*D*—H···*A*	*D*—H	H···*A*	*D*···*A*	*D*—H···*A*
N1—H1N···O1W^i^	0.91	2.27	2.939 (6)	130
N1—H1N···O2W^ii^	0.91	2.33	3.038 (6)	135
N1—H2N···O4^iii^	0.91	1.85	2.731 (7)	164
N1—H3N···O3^iv^	0.91	2.24	2.981 (6)	138
N2—H4N···O3^v^	0.88	2.21	3.069 (6)	166
N3—H5N···O1W^vi^	0.91	2.06	2.961 (6)	172
N3—H6N···O2W^vi^	0.91	1.94	2.844 (6)	170
N3—H7N···O3^iv^	0.91	1.98	2.835 (6)	156
O1w—H1W···O2	0.84	1.90	2.720 (5)	167
O1w—H2W···O2^vii^	0.84	1.99	2.803 (5)	162
O2w—H3W···O1	0.84	1.89	2.732 (6)	176
O2w—H4W···O2^vi^	0.84	1.92	2.751 (5)	171

Symmetry codes: (i) *x*−1, *y*−1, *z*; (ii) *x*, *y*−1, *z*; (iii) −*x*, −*y*, −*z*; (iv) *x*, *y*, *z*+1; (v) −*x*+1, −*y*, −*z*; (vi) −*x*+1, −*y*+1, −*z*+1;  (vii) −*x*+2, −*y*+1, −*z*+1.
